# Supplementary material for: Sialoglycan binding triggers spike opening in a human coronavirus
Source: Nature. 2023 Oct 4;624(7990):201–6. doi: 10.1038/s41586-023-06599-z (PMC10700143; doi:10.1038/s41586-023-06599-z)
Supplement: Supplementary file 1 — Supplementary Figs 1–17 and Supplementary Tables 1–5. [file 41586_2023_6599_MOESM1_ESM.pdf]

nature portfolio

https://doi.org/10.1038/s41586-023-06599-z

# Supplementary information

# Sialoglycan binding triggers spike opening in a human coronavirus

In the format provided by the
authors and unedited

Supplementary Materials for

# Sialoglycan binding triggers spike opening in a human coronavirus

Matti F. Pronker, Robert Creutznacher, Ieva Drulyte, Ruben J.G. Hulswit, Zeshi Li, Frank J.M. van Kuppeveld, Joost Snijder, Yifei Lang, Berend-Jan Bosch, Geert-Jan Boons, Martin Frank, Raoul J. de Groot and Daniel L. Hurdiss

Correspondence to: r.j.degroot@uu.nl and d.l.hurdiss@uu.nl

**This PDF file includes:**

Figs. S1 to S17
Tables S1 to S4

**Other Supplementary Materials for this manuscript include the following:**

Videos S1 to S7

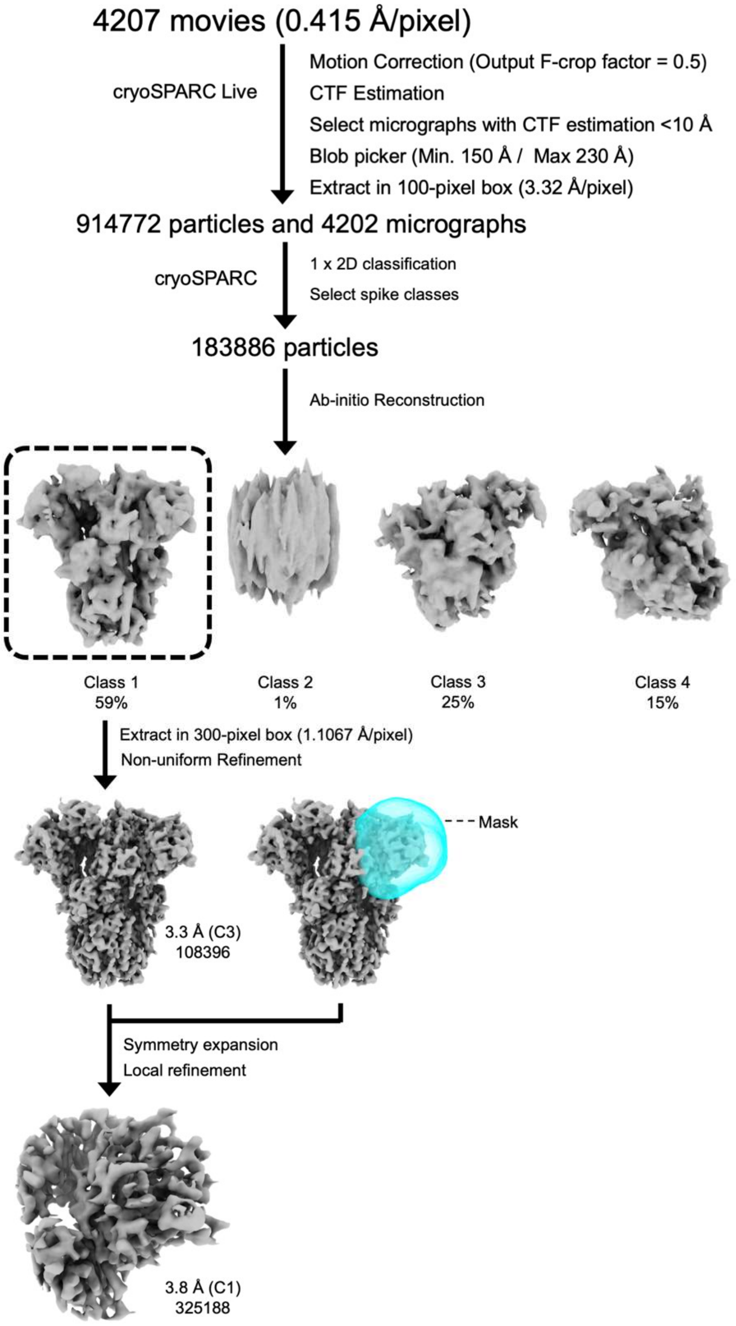


**Supplementary Fig. 1. Cryo-EM data processing pipeline for the *apo* HKU1-A spike glycoprotein.**

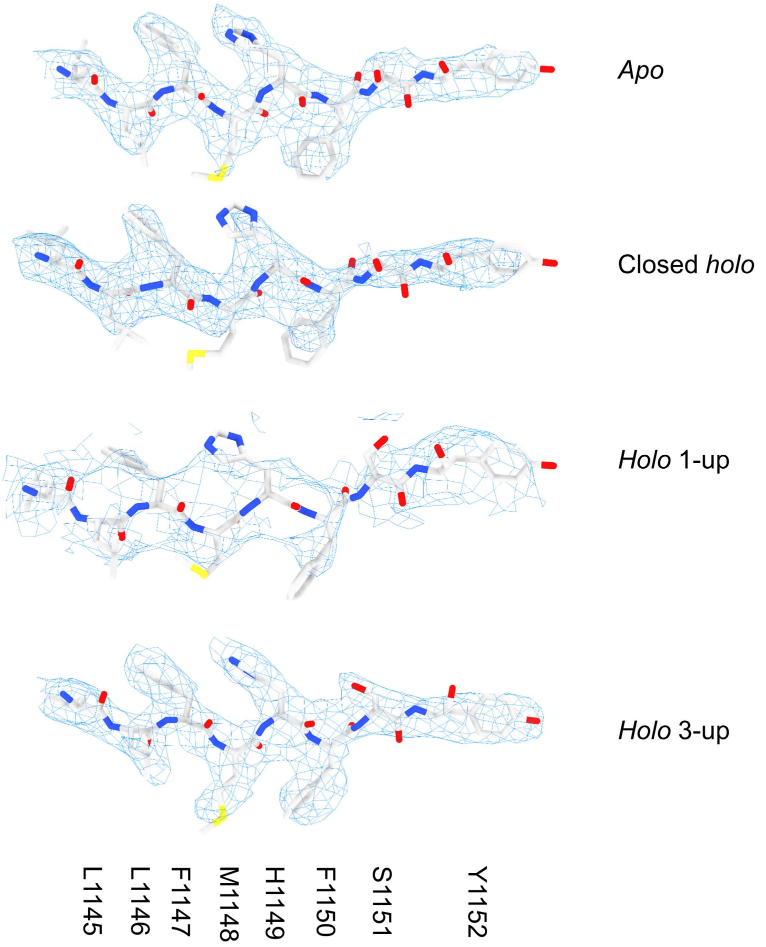


**Supplementary Fig. 2. Example density and model from the S2 region of each of the cryo-EM reconstructions generated from the *apo* and *holo* data sets.**

```
HKU1-A      MLLIIFILPTTLAVIGDFNCTNFAINDLNTTIPRISEYVVDVSYGLGTYYILDRVYLNTT  60
HKU1-B      MFLIIFILPTTLAVIGDFNCTNSFINDYNKTIPRISEDVVDVSLGLGTYYVLNRVYLNTT  60
            *:********************  *** *.******* ***** ******:*:*******

HKU1-A      ILFTGYFPKSGANFRDLSLKGTTKLSTLWYQKPFLSDFNNGIFSRVKNTKLYVNKTLYSE  120
HKU1-B      LLFTGYFPKSGANFRDLALKGSIYLSTLWYKPPFLSDFNNGIFSKVKNTKLYVNNTLYSE  120
            :****************:***:  ******: ************:*********:*****

HKU1-A      FSTIVIGSVFINNSYTIVVQPHNGVLEITACQYTMCEYPHTICKSIGSSRNESWHFDKSE  180
HKU1-B      FSTIVIGSVFVNTSYTIVVQPHNGILEITACQYTMCEYPHTVCKSKGSIRNESWHIDSSE  180
            **********:*.***********:****************:*** ** ******:*.**

HKU1-A      PLCLFKKNFTYNVSTDWLYFHFYQERGTFYAYYADSGMPTTFLFSLYLGTLLSHYYVLPL  240
HKU1-B      PLCLFKKNFTYNVSADWLYFHFYQERGVFYAYYADVGMPTTFLFSLYLGTILSHYYVMPL  240
            **************:************.******* **************:******:**

HKU1-A      TCNAISSNTDNETLQYWVTPLSKRQYLLKFDDRGVITNAVDCSSSFFSEIQCKTKSLLPN  300
HKU1-B      TCNAISSNTDNETLEYWVTPLSRRQYLLNFDEHGVITNAVDCSSSFLSEIQCKTQSFAPN  300
            **************:*******:*****:**::*************:*******:*: **

HKU1-A      TGVYDLSGFTVKPVATVHRRIPDLPDCDIDKWLNNFNVPSPLNWERKIFSNCNFNLSTLL  360
HKU1-B      TGVYDLSGFTVKPVATVYRRIPNLPDCDIDNWLNNVSVPSPLNWERRIFSNCNFNLSTLL  360
            *****************:****:*******:****..*********:*************

HKU1-A      RLVHTDSFSCNNFDESKIYGSCFKSIVLDKFAIPNSRRSDLQLGSSGFLQSSNYKIDTTS  420
HKU1-B      RLVHVDSFSCNNLDKSKIFGSCFNSITVDKFAIPNRRRDDLQLGSSGFLQSSNYKIDISS  420
            ****.*******:*:***:****:**.:******* **.****************** :*

HKU1-A      SSCQLYYSLPAINVTINNYNPSSWNRRYGFNNFNLSSHSVVYSRYCFSVNNTFCPCAKPS  480
HKU1-B      SSCQLYYSLPLVNVTINNFNPSSWNRRYGFGSFNLSSYDVVYSDHCFSVNSDFCPCADPS  480
            ********** :******:***********..*****:.**** :*****. *****.**

HKU1-A      FASSCKSHKPPSASCPIGTNYRSCESTTVLDHTDWCRCSCLPDPITAYDPRSCSQKKSLV  540
HKU1-B      VVNSCAKSKPPSAICPAGTKYRHCDLDTTLYVKNWCRCSCLPDPISTYSPNTCPQKKVVV  540
            ...** . ***** ** **:** *:  *.*  .:***********::*.*.:* *** :*

HKU1-A      GVGEHCAGFGVDEEKCGVLDGSYNVSCLCSTDAFLGWSYDTCVSNNRCNIFSNFILNGIN  600
HKU1-B      GIGEHCPGLGINEEKCGTQL--NHSSCFCSPDAFLGWSFDSCISNNRCNIFSNFIFNGIN  598
            *:**** *:*::*****.     : **:** *******:*:*:************:****

HKU1-A      SGTTCSNDLLQPNTEVFTDVCVDYDLYGITGQGIFKEVSAVYYNSWQNLLYDFNGNIIGF  660
HKU1-B      SGTTCSNDLLYSNTEISTGVCVNYDLYGITGQGIFKEVSAAYYNNWQNLLYDSNGNIIGF  658
            **********  ***: *.***:*****************.***.******* *******

HKU1-A      KDFVTNKTYNIFPCYAGRVSAAFHQNASSLALLYRNLKCSYVLNNISLATQP-YFDSYLG  719
HKU1-B      KDFLTNKTYTILPCYSGRVSAAFYQNSSSPALLYRNLKCSYVLNNISFISQPFYFDSYLG  718
            ***:*****.*:***:*******:**:** *****************: :** *******

HKU1-A      CVFNADNLTDYSVSSCALRMGSGFCVDYNSPSSSSSRRKRRSISASYRFVTFEPFNVSFV  779
HKU1 B      CVLNAVNLTSYSVSSCDLRMGSGFCIDYALPS---SRRKRRGISSPYRFVTFEPFNVSFV  775
            **:** ***.****** ********:**  **   ******.**: **************

HKU1-A      NDSIESVGGLYEIKIPTNFTIVGQEEFIQTNSPKVTIDCSLFVCSNYAACHDLLSEYGTF  839
HKU1-B      NDSVETVGGLFEIQIPTNFTIAGHEEFIQTSSPKVTIDCSAFVCSNYAACHDLLSEYGTF  835
            ***:*:****:**:*******.*:******.********* *******************

HKU1-A      CDNINSILDEVNGLLDTTQLHVADTLMQGVTLSSNLNTNLHFDVDNINFKSLVGCLGPHC  899
HKU1-B      CDNINSILNEVNDLLDITQLQVANALMQGVTLSSNLNTNLHSDVDNIDFKSLLGCLGSQC  895
            ********:***.*** ***:**::**************** *****:****:**** :*

HKU1-A      GSSSRSFFEDLLFDKVKLSDVGFVEAYNNCTGGSEIRDLLCVQSFNGIKVLPPILSESQI  959
HKU1-B      GSSSRSLLEDLLFNKVKLSDVGFVEAYNNCTGGSEIRDLLCVQSFNGIKVLPPILSETQI  955
            ******::*****:*******************************************:**

HKU1-A      SGYTTAATVAAMFPPWSAAAGIPFSLNVQYRINGLGVTMDVLNKNQKLIATAFNNALLSI  1019
HKU1-B      SGYTTAATVAAMFPPWSAAAGVPFSLNVQYRINGLGVTMDVLNKNQKLIANAFNKALLSI  1015
            *********************:****************************.***:*****
```

```
HKU1-A      QNGFSATNSALAKIQSVVNSNAQALNSLLQQLFNKFGAISSSLQEILSRLDALEAQVQID 1079
HKU1-B      QNGFTATNSALAKIQSVVNANAQALNSLLQQLFNKFGAISSSLQEILSRLDNLEAQVQID 1075
            ****:**************:******************************* ********

HKU1-A      RLINGRLTALNAYVSQQLSDISLVKLGAALAMEKVNECVKSQSPRINFCGNGNHILSLVQ 1139
HKU1-B      RLINGRLTALNAYVSQQLSDITLIKAGASRAIEKVNECVKSQSPRINFCGNGNHILSLVQ 1135
            *********************:*:* **: *:****************************

HKU1-A      NAPYGLLFMHFSYKPISFKTVLVSPGLCISGDVGIAPKQGYFIKHNDHWMFTGSSYYYPE 1199
HKU1-B      NAPYGLLFIHFSYKPTSFKTVLVSPGLCLSGDRGIAPKQGYFIKQNDSWMFTGSSYYYPE 1195
            ********:****** ************:*** ***********:** ************

HKU1-A      PISDKNVVFMNTCSVNFTKAPLVYLNHSVPKLSDFESELSHWFKNQTSIAPNLTLNLHTI 1259
HKU1-B      PISDKNVVFMNSCSVNFTKAPFIYLNNSIPNLSDFEAELSLWFKNHTSIAPNLTFNSH-I 1254
            ***********:*********::***:*:*:*****:*** ****:********:* * *

HKU1-A      NATFLDLYYEMNLIQESIKSLNNSYINLKDIGTYEMYVKWPWYVWLLISFSFIIFLVLLF 1319
HKU1-B      NATFLDLYYEMNVIQESIKSLNSSFINLKEIGTYEMYVKWPWYIWLLIVILFIIFLMILF 1314
            ************:*********.*:****:*************:**** : *****::**

HKU1-A      FICCCTGCGSACFSKCHNCCDEYGGHHDFVIKTSHDD                        1356
HKU1-B      FICCCTGCGSACFSKCHNCCDEYGGHNDFVIKASHDD                        1351
            **************************:*****:****
```

**Supplementary Fig. 3. Sequence alignment of HKU1-A and HKU1-B spike glycoproteins.**

Comparison of HKU1 S protein sequences from genotypes A (Caen1, GenBank entry ADN03339.1) and B (isolate N5, NCBI entry Q0ZME7) aligned using the ClustalW algorithm.

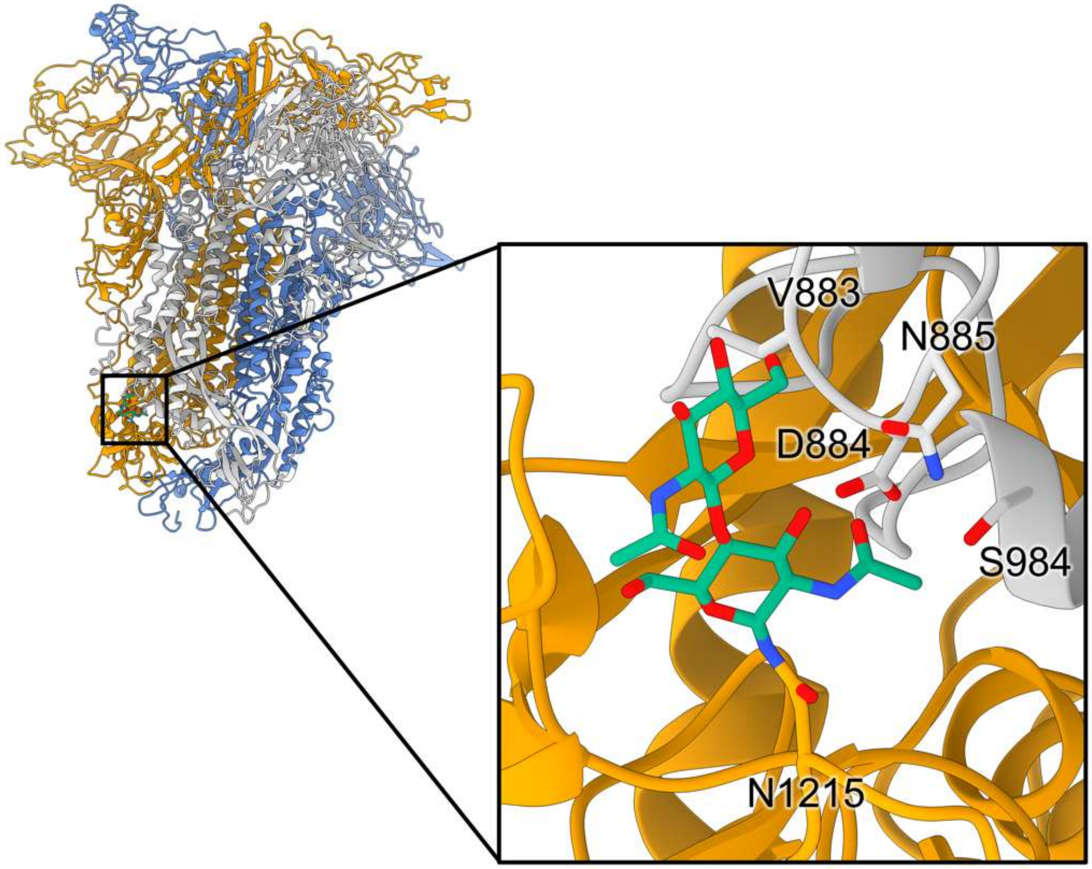


**Supplementary Fig. 4. *N*-linked glycosylation of N1215 stabilises the trimer at its base via contacts with the counter-clockwise neighbouring protomer.**

Residues from the neighbouring protomer (in grey) contacting this *N*-linked glycan (green) are indicated as sticks.

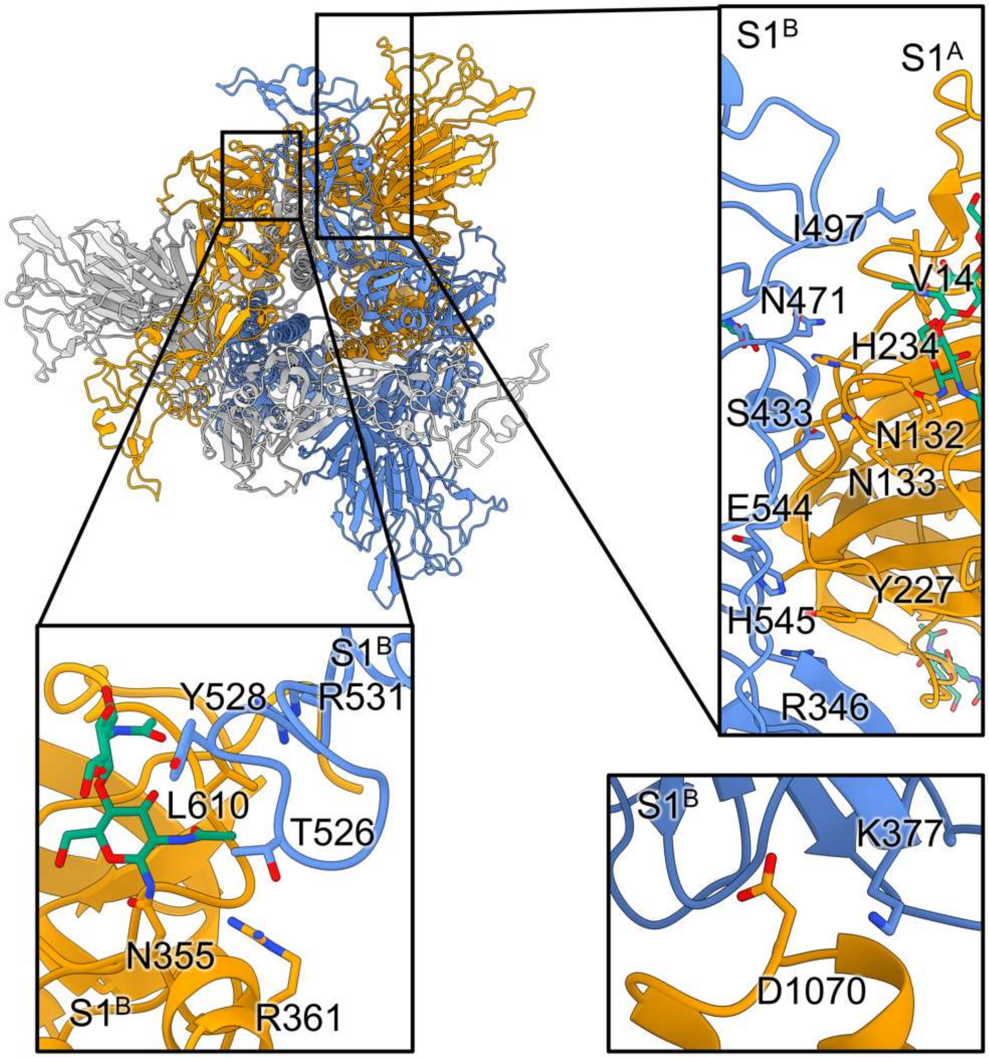


**Supplementary Fig. 5. Several minor interfaces stabilise the S1$^B$ domains in a downward orientation.** Several minor interfaces stabilise the S1$^B$ domains in a downward orientation in the *apo* state, such as S1$^B$-S1$^B$ (bottom left), S1$^B$-S1$^A$ (top right) and S1$^B$-S2 (bottom right). The S1$^B$-S1$^B$ interface (bottom left) is stabilised by an *N*-linked glycan on N355.

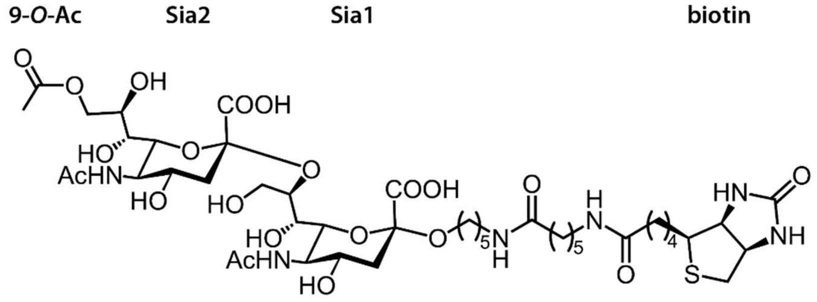


**Supplementary Fig. 6. Structure of the receptor analogue 9-*O*-Ac-Neu5Ac-α2,8-Neu5Ac-Lc-biotin**

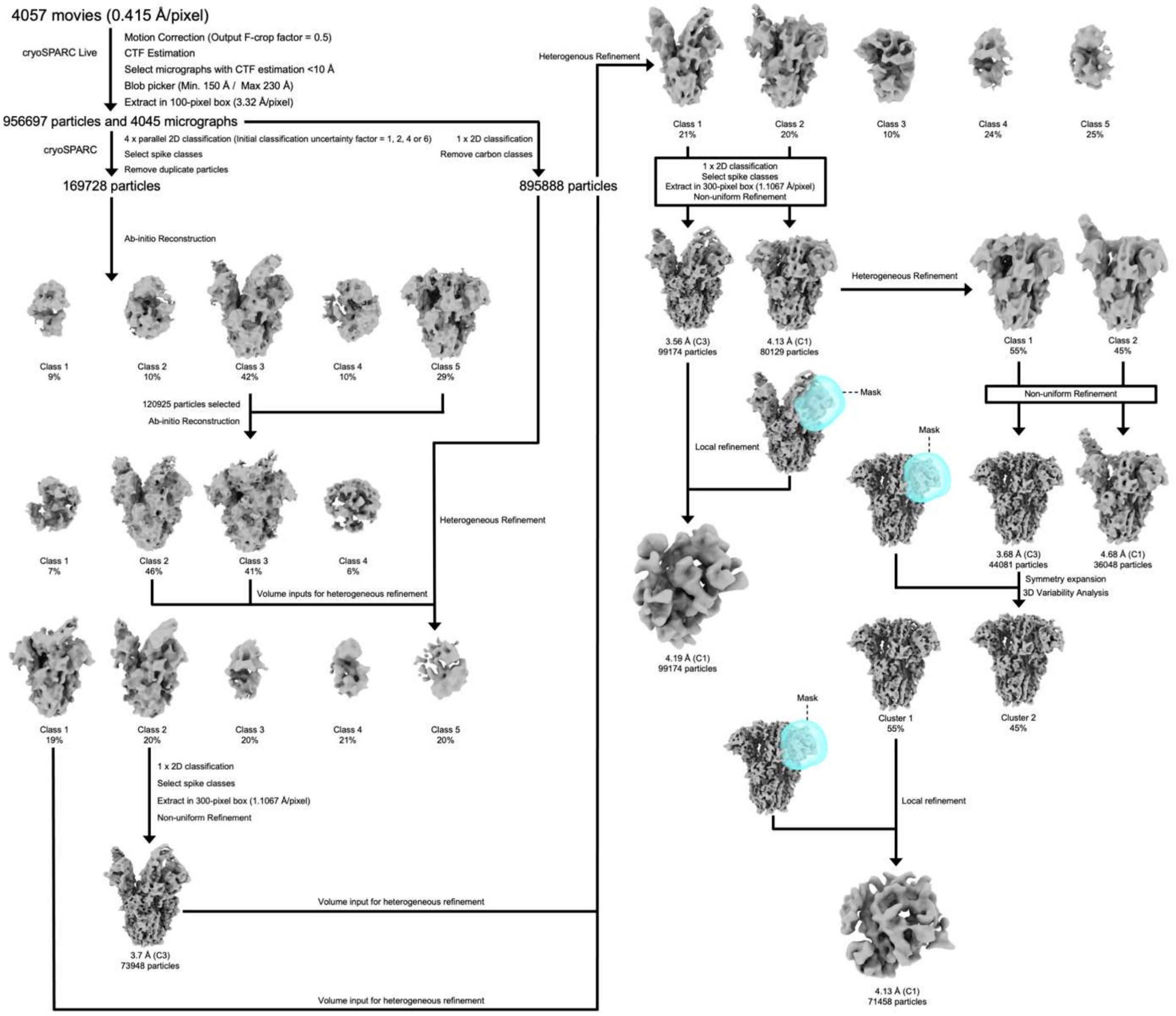


**Supplementary Fig. 7. Cryo-EM data processing pipeline for the *holo* HKU1-A spike glycoprotein.**

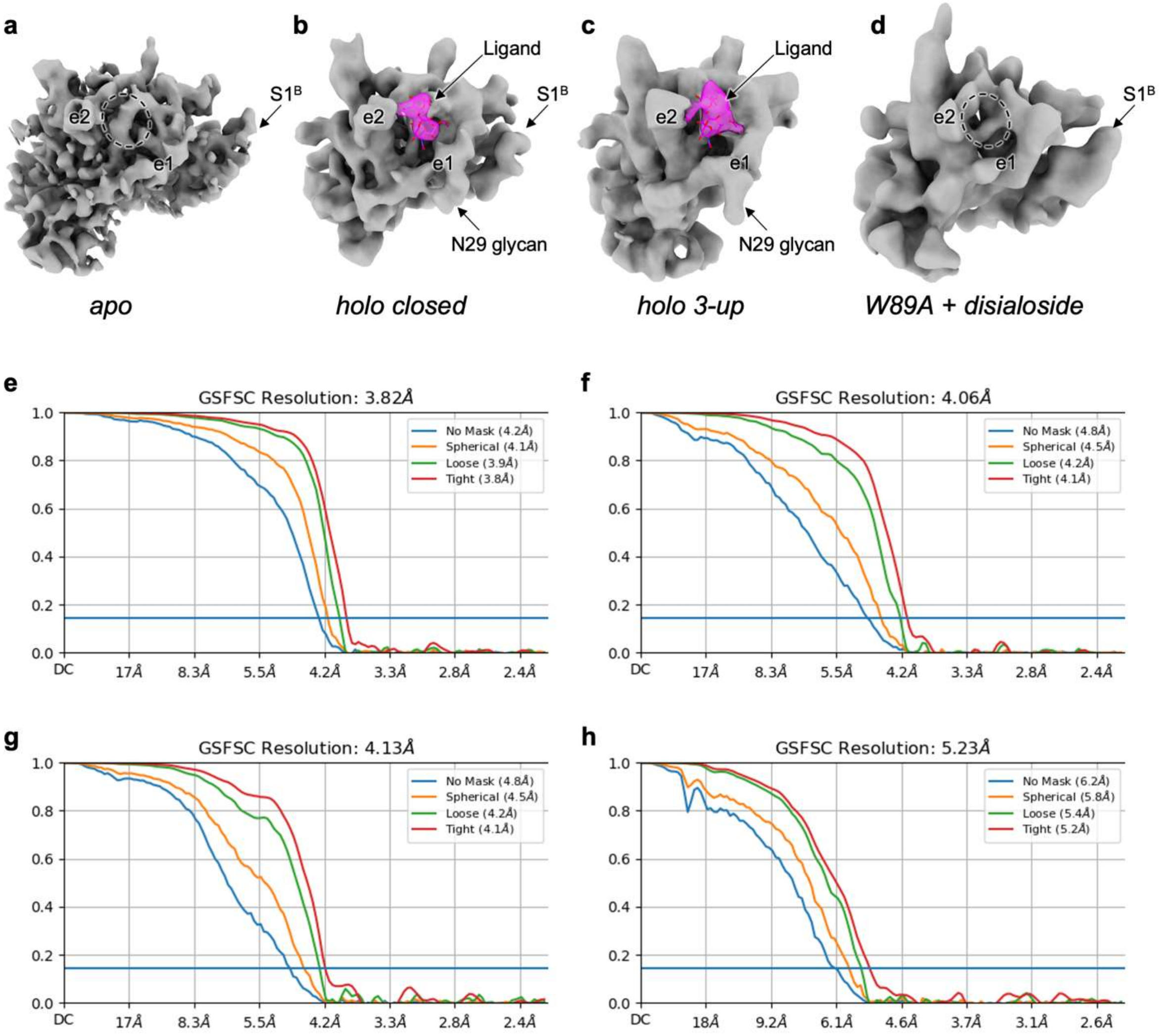


**Supplementary Fig. 8. Local refinements of the HKU1-A S1$^{A}$ domain.**
**a**, Locally refined maps of the *apo*, **b**, closed *holo*, **c**, *holo* 3-up and **d**, W89A mutant HKU1-A incubated with the disialoside. The spike protein is coloured grey and density for the disialoside, present only in the *holo* maps, is coloured magenta and contains the fitted coordinates for the molecule. In panels A and D, the receptor binding site is circled. **e**, Gold-standard FSC curves generated from the independent half maps contributing to the local refinements of the *apo*, **f**, closed *holo*, **g**, *holo* 3-up and **h**, W89A mutant HKU1-A incubated with the disialoside.

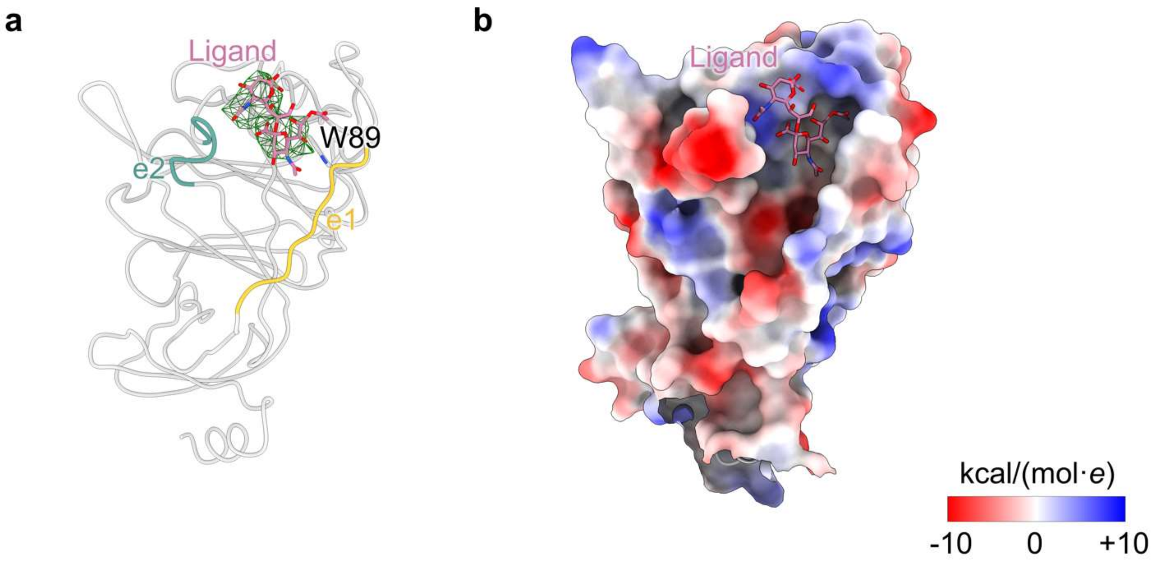


**Supplementary Fig. 9. Ligand binding site in the S1$^{A}$ domain.**

**a**, Difference density (closed *holo* minus *apo*) for the disialoside ligand bound to the S1$^{A}$ domain in the closed *holo* state confirms the binding site. **b**, Electrostatic potential map around the ligand binding site in the S1$^{A}$ domain shows a positively charged crevice in which the negatively charged disialoside binds.

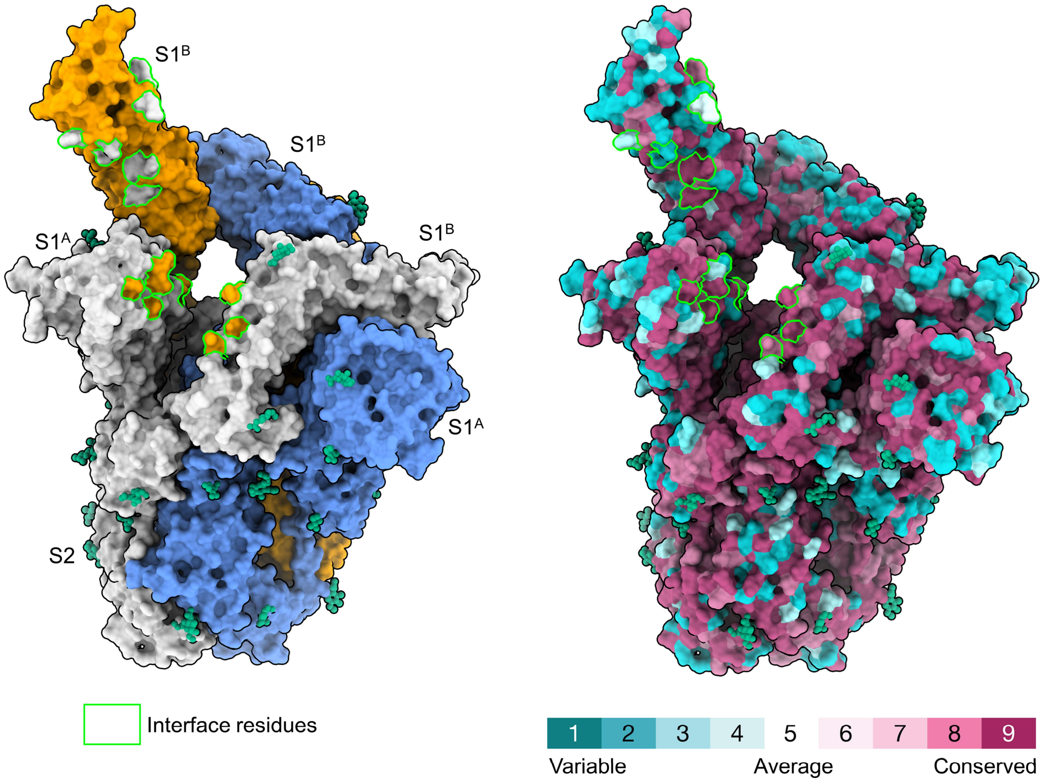


**Supplementary Fig. 10. Surface conservation of S1$^B$ occluded in the closed state.**
In the left panel, protomers of a 1-up HKU1-A S trimer are coloured grey, blue and orange and glycans are indicated in dark green. The footprint of residues contacting neighbouring S1$^A$ and S1$^B$ domains in the closed state, but becoming exposed upon S1$^B$ flipping up, are indicated in the colour of the protomer they were originally contacting, outlined in green. The same footprints are again outlined in green in the right panel, but on the 1-up S trimer in the same orientation with its surface coloured by evolutionary conservation (glycans still indicated in dark green).

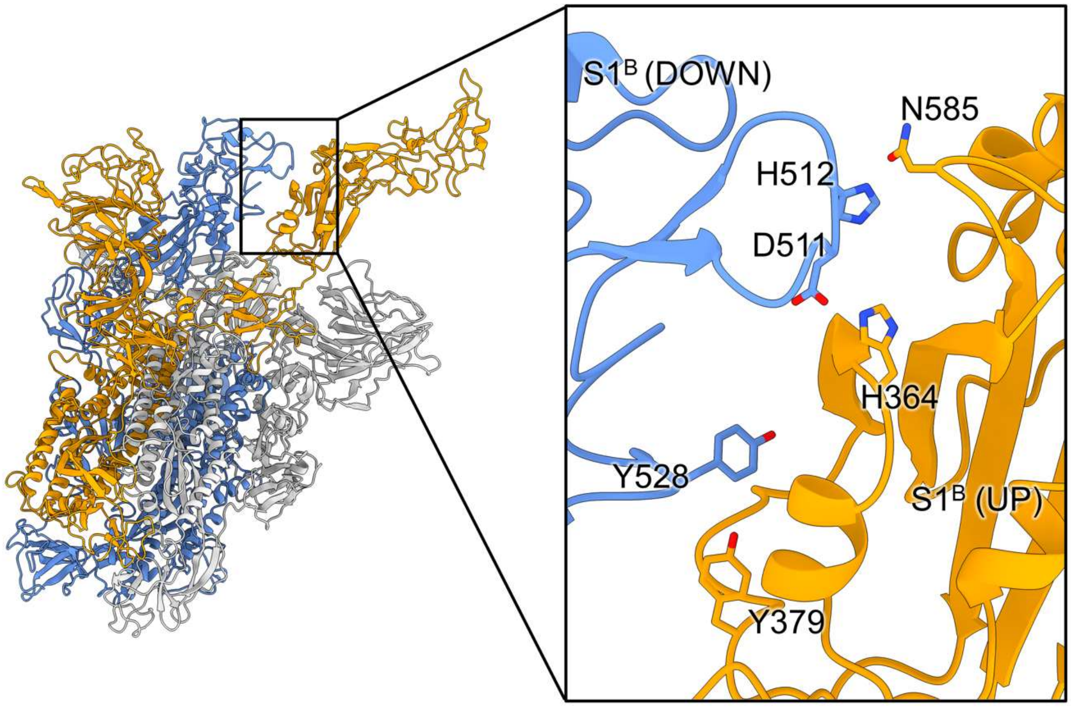


**Supplementary Fig. 11. A unique interface between the upward and downward S1$^{B}$ domains in the 1-up state.**

Residues at the interface are indicated, although the local resolution limits interpretability of side chain conformations.

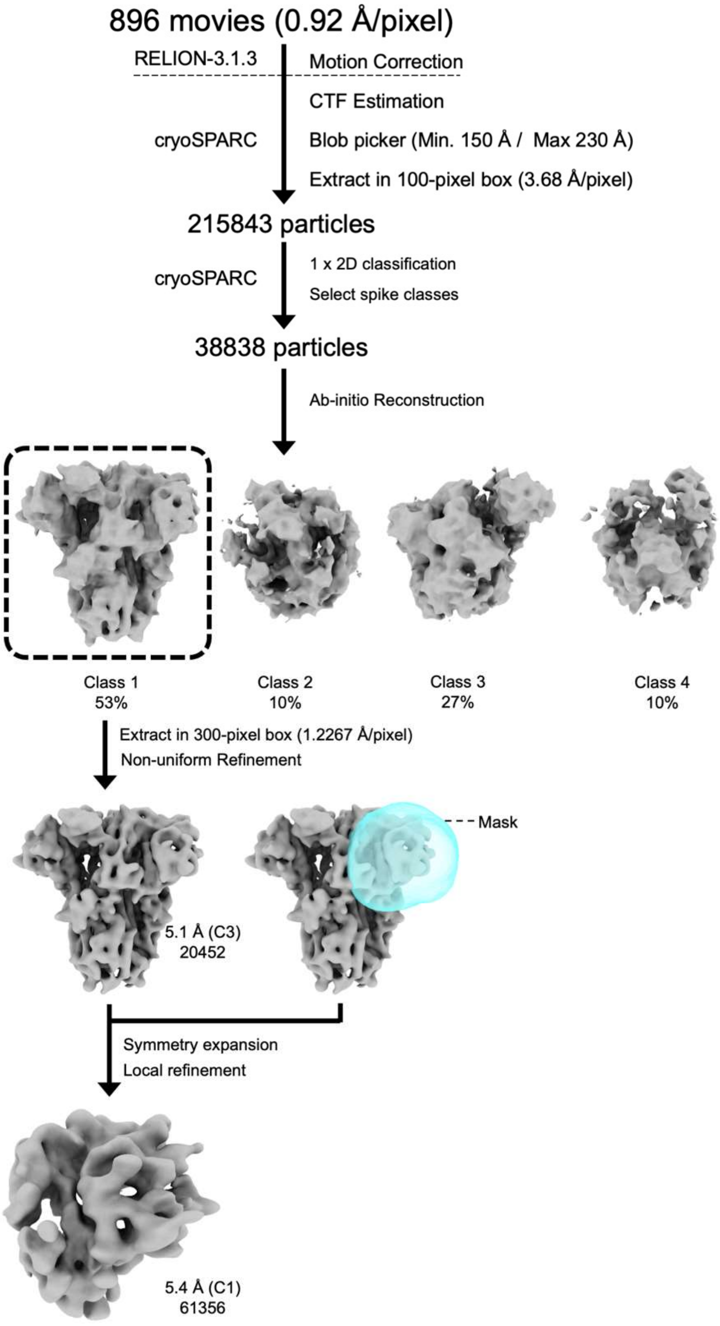


**Supplementary Fig. 12. Cryo-EM data processing pipeline for the W89A mutant HKU1-A spike glycoprotein.**

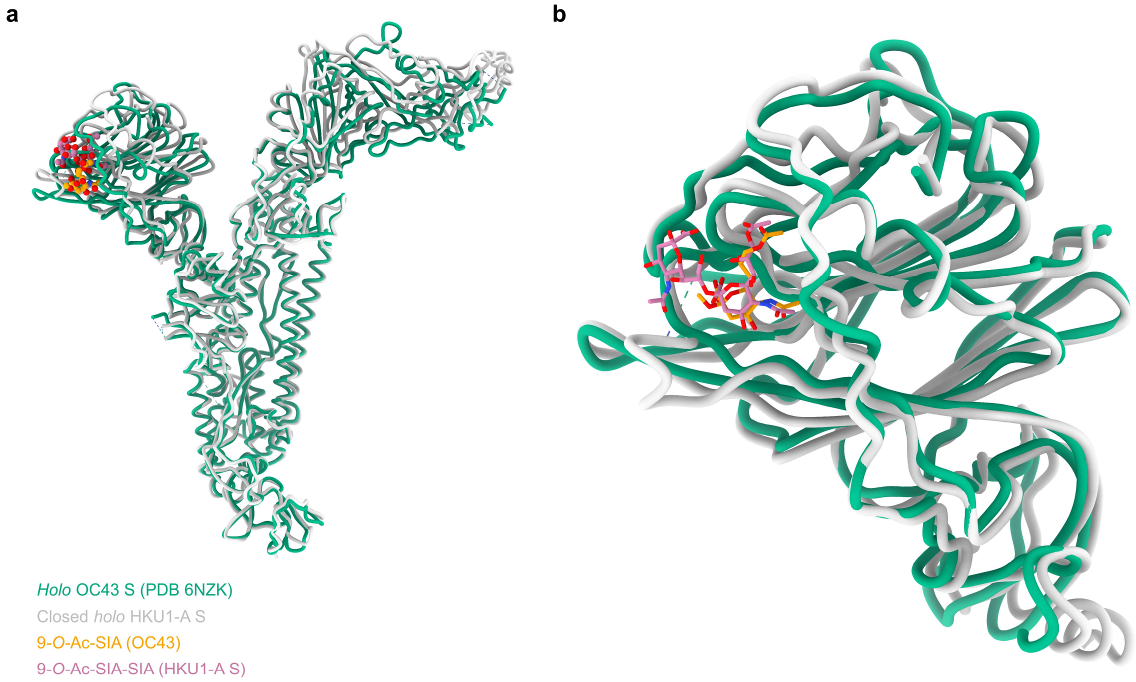


**Supplementary Fig. 13. Comparison of our closed *holo* HKU1-A S with the previously published *holo* OC43 S structure**

**a,** Comparison of a protomer of the OC43 S (green) bound with a 9-*O*-acetylated sialic acid (orange) with our closed *holo* $S1^A$, aligned on the S2 segment. **b**, Close-up comparison of the $S1^A$ domains of OC43 and our HKU1-A aligning on the $S1^A$ domain instead of the whole spike (same colouring as panel **b**).

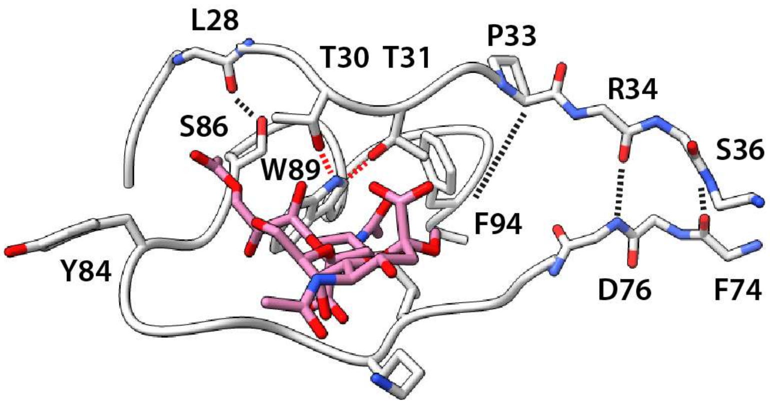


**Supplementary Fig. 14. Alternative arrangement of the disialoside binding pocket.**

The structure shown is based on MD-simulations of the HKU1-A N1 reference strain (GenBank entry NC_006577.2, Extended Data Fig. 10b). Notably, the essential hydrogen bond with W89 can be formed by the T30 backbone carbonyl (*cf*. Fig. 5a), or the T30 and T31 sidechains (red lines).

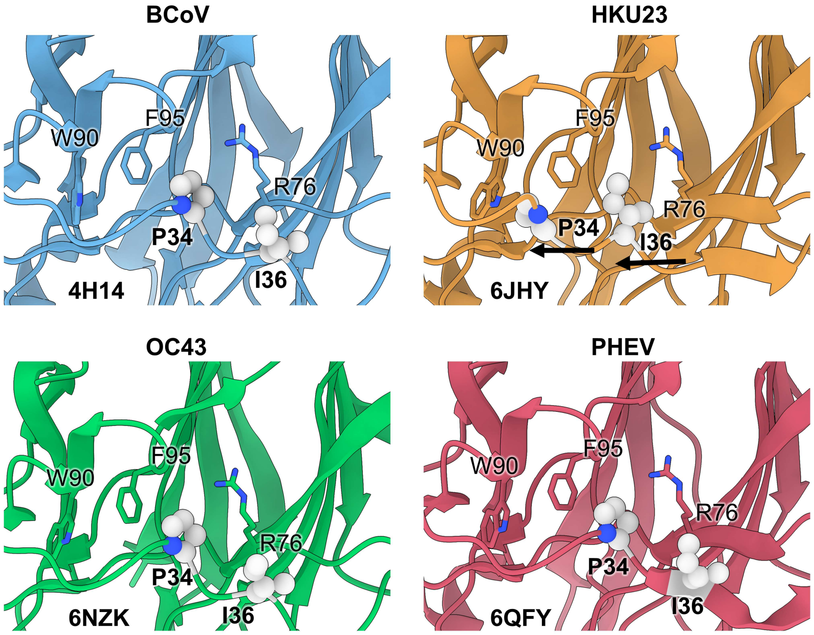


**Supplementary Fig. 15. The topology of the e1 loop in different CoVs observed in the PDB.** Shift of the e1 loop of HKU23 S (orange, top-right) compared to other coronavirus $S1^A$ domain e1 loops, exemplified by e1 residues P34 and I36 (light grey spheres). Side chains of W90, F95 and R76 are indicated as sticks for reference. The dromedary camel CoV HKU23 is the only CoV displaying a similar conformation of e1 as found in our HKU-1 *apo* structure. We note that for BCoV, HKU23 and PHEV structures, e1 residues are involved in crystal contacts which may artificially shift the equilibrium away from preferred conformations in a physiological setting.

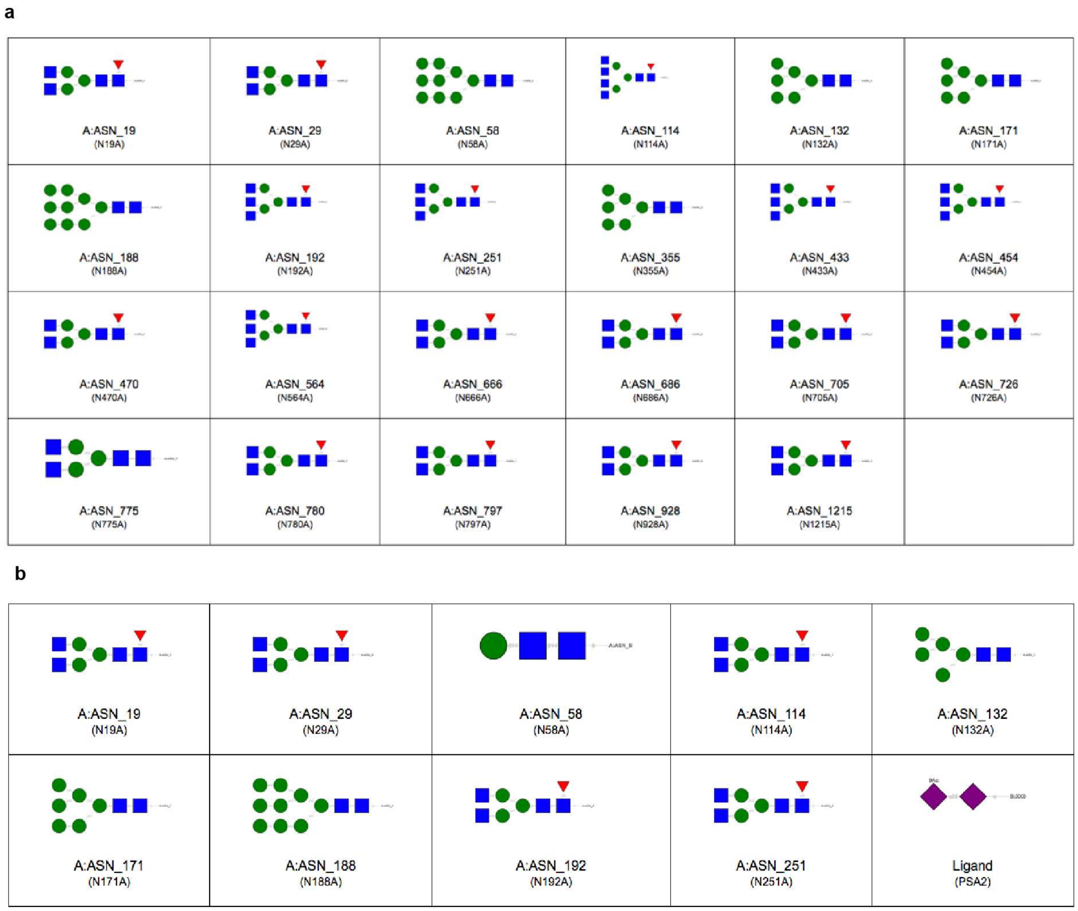


**Supplementary Fig. 16. N-glycans used in MD simulations of the HKU1-A spike ectodomain (a, see Extended Data Fig. 2) and S1$^{A}$ domain (b, see Extended Data Fig. 8-10)**

SNFG representations of the N-glycans were generated using Conformational Analysis Tools (CAT, http://www.md-simulations.de/CAT/).

Amino acid sequence of HKU1 CD5-SED-GCN4-Tx-ST

MPMGSLQPLATLYLLGMLVASVLAVIGDFNCTNFAINDLNTTIPRISEYVVDVSYGLGTYYILDRVYLNTTILFTGY
FPKSGANFRDLSLKGTTKLSTLWYQKPFLSDFNNGIFSRVKNTKLYVNKTLYSEFSTIVIGSVFINNSYTIVVQPHN
GVLEITACQYTMCEYPHTICKSIGSSRNESWHFDKSEPLCLFKKNFTYNVSTDWLYFHFYQERGTFYAYYADSGMPT
TFLFSLYLGTLLSHYYVLPLTCNAISSNTDNETLQYWVTPLSKRQYLLKFDDRGVITNAVDCSSSFFSEIQCKTKSL
LPNTGVYDLSGFTVKPVATVHRRIPDLPDCDIDKWLNNFNVPSPLNWERKIFSNCNFNLSTLLRLVHTDSFSCNNFD
ESKIYGSCFKSIVLDKFAIPNSRRSDLQLGSSGFLQSSNYKIDTTSSSCQLYYSLPAINVTINNYNPSSWNRRYGFN
NFNLSSHSVVYSRYCFSVNNTFCPCAKPSFASSCKSHKPPSASCPIGTNYRSCESTTVLDHTDWCRCSCLPDPITAY
DPRSCSQKKSLVGVGEHCAGFGVDEEKCGVLDGSYNVSCLCSTDAFLGWSYDTCVSNNRCNIFSNFILNGINSGTTC
SNDLLQPNTEVFTDVCVDYDLYGITGQGIFKEVSAVYYNSWQNLLYDFNGNIIGFKDFVTNKTYNIFPCYAGRVSAA
FHQNASSLALLYRNLKCSYVLNNISLATQPYFDSYLGCVFNADNLTDYSVSSCALRMGSGFCVDYNSPSSSSSGGSG
SSISASYRFVTFEPFNVSFVNDSIESVGGLYEIKIPTNFTIVGQEEFIQTNSPKVTIDCSLFVCSNYAACHDLLSEY
GTFCDNINSILDEVNGLLDTTQLHVADTLMQGVTLSSNLNTNLHFDVDNINFKSLVGCLGPHCGSSSRSFFEDLLFD
KVKLSDVGFVEAYNNCTGGSEIRDLLCVQSFNGIKVLPPILSESQISGYTTAATVAAMFPPWSAAAGIPFSLNVQYR
INGLGVTMDVLNKNQKLIATAFNNALLSIQNGFSATNSALAKIQSVVNSNAQALNSLLQQLFNKFGAISSSLQEILS
RLDALEAQVQIDRLINGRLTALNAYVSQQLSDISLVKLGAALAMEKVNECVKSQSPRINFCGNGNHILSLVQNAPYG
LLFMHFSYKPISFKTVLVSPGLCISGDVGIAPKQGYFIKHNDHWMFTGSSYYYPEPISDKNVVFMNTCSVNFTKAPL
VYLNHSVPKLSDFESELSHWFKNQTSIAPNLTLNLHTINATFLDLLIKRMKQIEDKIEEIESKQKKIENEIARIKKI
KLVPRGSLEWSHPQFEK*

Coding sequence HKU1 CD5-SED-GCN4-Tx-ST

**ATGCCCATGGGGTCTCTGCAACCGCTGGCCACCTTGTACCTGCTGGGGATGCTGGTCGCTTCCGTGCTA**gcaGTTAT
AGGTGATTTTAATTGTACTAATTTTGCTATTAATGATTTAAACACCACAATTCCTCGCATAAGTGAGTATGTTGTGG
ATGTTTCTTATGGTTTGGGTACATATTATATACTTGATCGTGTTTATTTAAATACTACTATATTATTTACTGGTTAT
TTCCCTAAATCTGGTGCCAATTTTAGGGATCTATCTTTAAAAGGTACTACAAAATTGAGTACTCTTTGGTATCAGAA
ACCCTTTTTATCTGATTTTAATAATGGTATTTTTTCTAGAGTTAAGAATACTAAGTTGTATGTTAATAAAACTTTGT
ATAGTGAGTTTAGTACTATAGTTATAGGTAGTGTTTTTATTAACAACTCTTATACTATTGTTGTTCAACCTCATAAT
GGTGTTTTGGAGATTACAGCTTGTCAATACACTATGTGTGAGTATCCTCATACTATTTGTAAATCTATAGGTAGTTC
TCGTAATGAATCTTGGCATTTTGATAAATCTGAACCTTTGTGTCTGTTCAAGAAAAATTTTACTTATAATGTTTCTA
CAGATTGGTTGTATTTTCATTTTTATCAAGAACGTGGCACTTTTTATGCTTATTATGCTGATTCTGGCATGCCTACT
ACTTTTTTATTTAGTTTGTATCTTGGTACTCTTTTATCTCATTATTATGTTTTGCCTTTGACTTGTAATGCTATATC
TTCTAATACTGATAATGAGACTTTACAATATTGGGTCACACCTTTGTCTAAACGCCAATATCTTCTTAAATTTGACG
ACCGTGGTGTTATTACTAATGCTGTTGATTGTTCTAGTAGTTTCTTTAGCGAGATTCAATGTAAAACTAAATCTTTA
TTACCTAATACTGGTGTTTATGACTTATCTGGTTTTACTGTTAAGCCTGTTGCAACTGTACATCGTCGTATTCCTGA
TTTACCTGATTGTGACATTGATAAATGGCTTAACAATTTTAATGTACCCTCACCTCTTAATTGGGAACGTAAAATTT
TTTCTAATTGCAACTTTAATTTGAGTACTTTGCTTCGTTTAGTTCATACTGATTCTTTTTCTTGTAATAATTTTGAT
GAATCTAAGATATATGGTAGTTGTTTTAAGAGTATTGTTTTAGATAAATTTGCCATACCCAACTCCAGACGATCTGA
TTTGCAGTTGGGCAGTTCTGGTTTTCTGCAATCTTCTAATTATAAAATTGACACTACTTCTAGTTCTTGTCAATTGT
ATTATAGTTTGCCTGCAATTAATGTTACTATTAATAATTATAATCCTTCTTCTTGGAATAGAAGGTATGGTTTTAAT
AATTTTAATTTGAGTTCTCATAGTGTTGTTTACTCACGTTATTGTTTTTCTGTTAATAATACTTTTTGTCCTTGTGC
TAAACCTTCTTTTGCTTCAAGTTGCAAGAGTCATAAACCACCTTCTGCTTCTTGTCCTATTGGTACTAATTATCGTT
CTTGTGAGAGTACTACTGTACTCGACCACACTGACTGGTGTAGGTGTTCTTGTTTACCTGATCCTATAACTGCTTAT
GACCCTAGGTCTTGTTCTCAAAAAAAGTCTCTGGTTGGTGTTGGTGAACATTGTGCAGGGTTCGGTGTTGATGAAGA
AAAGTGTGGTGTATTGGATGGATCATATAATGTTTCTTGTCTTTGTAGTACTGATGCCTTTCTAGGTTGGTCTTATG
ACACTTGCGTCAGTAACAACCGTTGTAATATTTTTTCTAATTTTATTTTAAATGGTATCAATAGTGGTACCACTTGT
TCTAATGATTTATTGCAGCCTAATACTGAAGTTTTTACTGATGTTTGTGTTGATTACGACCTTTATGGTATTACAGG
ACAAGGTATTTTTAAAGAAGTTTCTGCTGTTTATTATAATAGTTGGCAAAATCTTTTGTATGATTTTAATGGCAACA
TTATTGGTTTTAAAGATTTTGTTACTAATAAAACATATAATATTTTCCCTTGTTATGCAGGAAGAGTTTCTGCTGCT
TTTCATCAAAATGCTTCCTCTTTGGCTTTACTTTATCGTAATTTAAAATGTAGCTATGTTTTGAATAATATTTCTTT
AGCTACTCAGCCATATTTTGATAGTTATCTTGGTTGCGTTTTTAATGCTGATAATTTAACTGATTATTCTGTTTCTT
CTTGTGCTCTTCGCATGGGTAGTGGTTTTTGTGTTGATTATAACTCACCTTCTTCTTCCTCTTCGGGTGGTTCTGGT
TCTAGTATTTCTGCTTCTTATCGGTTTGTTACTTTTGAACCCTTTAATGTCAGTTTTGTTAATGACAGTATTGAGTC
TGTGGGTGGTCTTTATGAGATCAAAATTCCCACTAACTTTACTATAGTTGGTCAAGAGGAATTTATTCAAACTAATT

CTCCTAAAGTTACTATTGATTGTTCTTTATTTGTCTGTTCTAATTATGCAGCTTGCCATGACTTATTGTCAGAGTAT
GGCACTTTTTGTGATAATATTAATAGTATTTTAGATGAAGTTAATGGTTTACTTGATACTACTCAATTGCATGTAGC
TGATACTCTTATGCAAGGTGTCACACTTAGCTCCAATCTTAATACTAATTTGCATTTTGATGTTGATAATATTAATT
TTAAATCCCTAGTTGGATGTTTAGGTCCACACTGCGGTTCTTCTTCTCGTTCTTTTTTTGAAGATTTATTGTTTGAC
AAAGTTAAACTTTCAGATGTTGGTTTTGTTGAAGCTTATAACAATTGTACTGGTGGTAGTGAAATTAGAGATCTTCT
TTGTGTACAATCCTTTAATGGTATTAAAGTTTTGCCTCCTATTTTGTCTGAATCTCAAATTTCTGGTTACACCACAG
CCGCTACTGTTGCTGCTATGTTTCCACCATGGTCAGCAGCAGCTGGCATACCATTTTCTCTTAATGTACAATATAGA
ATTAATGGTTTGGGTGTTACTATGGATGTTCTTAATAAAAATCAAAAGTTGATAGCTACTGCTTTTAATAATGCTCT
TCTTTCTATTCAGAATGGTTTTAGTGCTACCAACTCTGCACTTGCTAAAATACAAAGTGTTGTTAATTCTAATGCTC
AAGCACTTAATAGTTTGTTACAGCAATTATTTAATAAATTTGGTGCAATTAGTTCTTCTTTACAAGAAATTTTATCT
CGTCTCGATGCTTTAGAGGCTCAGGTTCAGATTGATAGGCTTATTAATGGTCGTTTAACTGCTTTAAATGCTTATGT
TTCTCAACAGCTTAGTGATATTTCTCTTGTAAAACTTGGTGCTGCTTTAGCTATGGAGAAGGTTAATGAGTGTGTTA
AAAGTCAATCTCCTCGTATTAATTTTTGTGGTAATGGTAATCATATTTTGTCATTAGTTCAAAATGCTCCTTATGGT
TTGTTGTTTATGCATTTTAGTTATAAACCTATTTCTTTTAAAACTGTTTTAGTAAGTCCTGGTTTATGTATATCAGG
TGATGTAGGTATTGCACCTAAACAAGGGTATTTTATTAAACATAATGATCATTGGATGTTTACTGGTAGTTCTTACT
ATTATCCTGAACCAATTTCAGATAAAAATGTTGTTTTTATGAATACTTGTTCTGTTAATTTTACTAAAGCGCCTCTT
GTTTATTTGAATCATTCTGTACCAAAATTGTCTGATTTTGAATCTGAGTTATCTCATTGGTTTAAAAATCAAACATC
CATTGCGCCTAATTTGACTTTAAATCTTCATACTATTAATGCTACTTTTTTAGATTTGtta**ATTAAgCGCATGAAGC**
**AGATCGAGGACAAGATCGAAGAGATCGAGTCCAAGCAGAAGAAGATCGAGAACGAGATCGCCCGCATCAAGAAGatt**
**aagctggtgccgcgcggcagcctcgagtggagccacccgcagttcgagaag**tga

**Supplementary Fig. 17. HKU1-A S-ectodomain construct used in this study.**

The signal peptide (yellow), GCN4 trimerization domain (green), thrombin cleavage site (purple) and Strep-Tag (red) are indicated.

**Supplementary Table 1. Summary of coronavirus spike proteins determined by cryo-EM and their $S1^B$ state.**

| Subgenus | Virus | PDB ID | Reference | $S1^B$ up? |
|---|---|---|---|---|
| **Alpha** | | | | |
| *Duvinacovirus* | HCoV-229E | 6U7H, 7CYC | 10.7554/eLife.51230[1]<br>10.1038/s41467-020-20401-y[2] | **No** |
| *Setracovirus* | HCoV-NL63 | 5SZS | 10.1038/nsmb.3293[3] | **No** |
| *Tegacovirus* | FIPV | 6JX7 | 10.1073/pnas.1908898117[4] | **No** |
| | CCoV-HuPn-2018 | 7U0L, 7US6, 7US9, 7USA, 7USB | 10.1016/j.cell.2022.05.019[5] | **No** |
| *Rhinacovirus* | HKU2 | 6M15 | 10.1038/s41467-020-16876-4[6] | **No** |
| | SADS-CoV | 6M39 | 10.1128/JVI.01301-20[7] | **No** |
| *Pedacovirus* | PEDV | 6U7K, 6VV5, 7W6M, 7W73, 7Y6S, 7Y6T, 7Y6U, 7Y6V | 10.1038/s41467-022-32588-3[8]<br>10.1016/j.str.2020.12.003[9]<br>10.1128/JVI.00923-19[10] | ***Yes*** |
| **Beta** | | | | |
| *Embecovirus* | MHV | 3JCL, 6VSJ | 10.1038/nature16988[11]<br>10.1371/journal.ppat.1008392[12] | **No** |
| | HCoV-HKU1-B | 5I08 | 10.1038/nature17200[13] | **No** |
| | HCoV-OC43 | 6OHW, 6NZK, 7SB3 | 10.1038/s41594-019-0233-y[14]<br>10.1126/sciadv.abn2911[15] | **No** |
| *Sarbecovirus* | SARS-CoV | 5X5B, 5X58 | 10.1038/ncomms15092[16] | ***Yes*** |
| | SARS-CoV-2 | 6VXX, 6VYB | 10.1016/j.cell.2020.02.058[17] | ***Yes*** |
| | Pangolin sarbecovirus | 7BBH, 7CN8 | 10.1038/s41467-021-21006-9[18]<br>10.1038/s41467-021-21767-3[19] | **No** |
| | RaTG13 | 6ZGF | 10.1038/s41594-020-0468-7[20] | **No** |
| *Merbecovirus* | MERS-CoV | 5X59, 5X5F | 10.1038/ncomms15092[16] | ***Yes*** |
| | PDF-2180 | 7U6R | 10.1038/s41586-022-05513-3[21] | **No** |
| **Gamma** | | | | |
| *Igacovirus* | IBV | 6CV0 | 10.1371/journal.ppat.1007009[22] | **No** |
| **Delta** | | | | |
| *Buldecovirus* | PDCoV | 6BFU, 6B7N | 10.1128/JVI.01628-17[23]<br>10.1128/JVI.01556-17[24] | **No** |

## Supplementary Table 2. Cryo-EM data collection, refinement and validation statistics for global and local refinements.

| | *apo* (EMDB-16882) (PDB 8OHN) | closed *holo* (EMDB-17076) (PDB 8OPM) | *holo* 1-up (EMDB-17077) (PDB 8OPN) | *holo* 3-up (EMDB-17078) (PDB 8OPO) | *holo* mutant W89A (EMDB-17079) | *apo* local $S1^A$ (EMDB-17080) | closed *holo* local $S1^A$ (EMDB-17081) | *holo* 3-up local $S1^A$ (EMDB-17082) | *holo* mutant local $S1^A$ W89A (EMDB-17083) |
|---|---|---|---|---|---|---|---|---|---|
| **Data collection and processing** | | | | | | | | | |
| Magnification | 105,000x | 105,000x | 105,000x | 105,000x | 150,000x | 105,000x | 105,000x | 105,000x | 150,000x |
| Voltage (kV) | 300 | 300 | 300 | 300 | 200 | 300 | 300 | 300 | 200 |
| Electron exposure (e–/Å$^2$) | 46.3 | 46.3 | 46.3 | 46.3 | 41.7 | 46.3 | 46.3 | 46.3 | 41.7 |
| Defocus range (μm) | 1.5-2.5 | 1.5-2.5 | 1.5-2.5 | 1.5-2.5 | 1.5-2.5 | 1.5-2.5 | 1.5-2.5 | 1.5-2.5 | 1.5-2.5 |
| Pixel size (Å) | 0.415* | 0.415* | 0.415* | 0.415* | 0.92 | 0.415* | 0.415* | 0.415* | 0.92 |
| Symmetry imposed | C3 | C3 | C1 | C3 | C3 | C1 | C1 | C1 | C1 |
| Initial particle images (no.) | 914772 | 956697 | 956697 | 956697 | 215843 | 914772 | 956697 | 956697 | 215843 |
| Final particle images (no.) | 108396 | 44081 | 36048 | 99174 | 38838 | 108396 | 71458 | 99174 | 61356 |
| Map resolution (Å) | 3.4 | 3.8 | 5 | 3.7 | 5.3 | 3.8 | 4.1 | 4.1 | 5.2 |
| FSC threshold | 0.143 | 0.143 | 0.143 | 0.143 | 0.143 | 0.143 | 0.143 | 0.143 | 0.143 |
| Map resolution range (Å) | 2.4-11 | 3.2-13 | 4.1-17 | 2.4-12 | 4.6-12.7 | 3.2-12.5 | 3.5-9 | 3.5-9.2 | 4.6-16.4 |
| **Refinement** | | | | | | | | | |
| Initial model used (PDB code) | 5KWB, 6NZK | 5KWB, 6NZK | 5KWB, 6NZK | 5KWB, 6NZK | - | - | - | - | - |
| Model resolution (Å) FSC threshold 0.5 | 3.7 | 4.1 | 6.0 | 4.1 | - | - | - | - | - |
| Map sharpening *B* factor (Å$^2$) | 105 | 165 | 300 | 55 | - | - | - | - | - |
| Model composition | | | | | | | | | |
| Non-hydrogen atoms | 29328 | 29802 | 28543 | 28965 | - | - | - | - | - |
| Protein residues | 3585 | 3582 | 3555 | 3585 | - | - | - | - | - |
| Ligands | 87 | 123 | 40 | 57 | - | - | - | - | - |
| *B* factors (Å$^2$) | | | | | | | | | |
| Protein | 113.8 | 171.1 | 368.1 | 28.9 | - | - | - | - | - |
| Ligand | 143.2 | 226.1 | 396.4 | 63.1 | - | - | - | - | - |
| R.m.s. deviations | | | | | | | | | |
| Bond lengths (Å) | 0.003 | 0.004 | 0.004 | 0.003 | - | - | - | - | - |
| Bond angles (°) | 0.65 | 0.82 | 0.91 | 0.82 | - | - | - | - | - |
| Validation | | | | | | | | | |
| MolProbity score | 1.51 | 1.84 | 1.86 | 1.67 | - | - | - | - | - |
| Clashscore | 3.94 | 6.42 | 7.68 | 5.52 | - | - | - | - | - |
| Poor rotamers (%) | 0.00 | 0.00 | 0.00 | 0.00 | - | - | - | - | - |
| Ramachandran plot | | | | | | | | | |
| Favored (%) | 95.30 | 91.90 | 93.27 | 94.65 | - | - | - | - | - |
| Allowed (%) | 4.70 | 7.93 | 6.59 | 5.26 | - | - | - | - | - |
| Disallowed (%) | 0.00 | 0.17 | 0.14 | 0.08 | - | - | - | - | - |

*Super-resolution pixel size

**Supplementary Table 3. HKU1 Caen1 S1$^{A}$-ligand hydrogen bond analysis.**

| Index | Donor | Acceptor | Population | Distance | Angle |
|---|---|---|---|---|---|
| 1 | B:SIA_2:N5(HN5) | A:LYS_80:O | 96.9 | 2.86 | 156.9 |
| 2 | A:THR_30:OG1(HG1) | B:SIA_2:O10 | 80.3 | 2.82 | 159.9 |
| 3 | A:THR_82:OG1(HG1) | B:SIA_2:O1B | 58.0 | 2.75 | 157.5 |
| 4 | B:SIA_1:N5(HN5) | A:THR_82:OG1 | 56.0 | 2.99 | 157.4 |
| 5 | A:THR_82:OG1(HG1) | B:SIA_2:O1A | 49.8 | 2.76 | 158.5 |
| 6 | A:ASN_26:ND2(HD21) | B:9AC_3:OA9 | 46.8 | 2.97 | 158.9 |
| 7 | A:SER_246:OG(HG) | B:SIA_2:O1A | 23.0 | 2.68 | 160.5 |
| 8 | B:SIA_1:O7(HO7) | A:SER_246:O | 21.1 | 2.77 | 161.0 |
| 9 | A:LYS_80:NZ(HZ3) | B:SIA_2:O1A | 18.3 | 2.85 | 151.7 |
| 10 | A:LYS_80:NZ(HZ2) | B:SIA_2:O1A | 18.1 | 2.84 | 151.2 |
| 11 | A:LYS_80:NZ(HZ3) | B:SIA_2:O1B | 18.0 | 2.86 | 148.5 |
| 12 | A:LYS_80:NZ(HZ2) | B:SIA_2:O1B | 16.9 | 2.84 | 149.4 |
| 13 | A:LYS_80:NZ(HZ1) | B:SIA_2:O1B | 16.5 | 2.84 | 150.3 |
| 14 | A:LYS_80:NZ(HZ1) | B:SIA_2:O1A | 15.4 | 2.84 | 152.1 |
| 15 | A:SER_246:OG(HG) | B:SIA_2:O1B | 14.5 | 2.68 | 159.9 |
| 16 | A:THR_82:N(H) | B:SIA_2:O1B | 9.5 | 2.90 | 144.6 |
| 17 | A:ASN_26:ND2(HD22) | B:9AC_3:OA9 | 6.9 | 2.91 | 157.4 |
| 18 | A:THR_82:N(H) | B:SIA_2:O8 | 6.9 | 3.03 | 154.8 |
| 19 | A:THR_82:N(H) | B:SIA_2:O1A | 6.5 | 2.90 | 146.0 |
| 20 | A:SER_246:OG(HG) | B:SIA_1:O10 | 5.2 | 2.76 | 158.8 |
| 21 | B:SIA_1:O7(HO7) | A:SER_246:OG | 4.6 | 2.91 | 151.7 |
| 22 | A:LYS_84:NZ(HZ1) | B:SIA_1:O1A | 4.5 | 2.86 | 151.3 |
| 23 | B:SIA_1:O9(HO9) | A:ASN_248:OD1 | 3.9 | 2.80 | 153.8 |
| 24 | A:LYS_84:NZ(HZ2) | B:SIA_1:O1A | 3.8 | 2.82 | 155.3 |
| 25 | A:LYS_84:NZ(HZ1) | B:SIA_1:O1B | 3.8 | 2.82 | 155.3 |
| 26 | A:LYS_84:NZ(HZ3) | B:SIA_1:O1A | 3.8 | 2.82 | 155.6 |
| 27 | A:LYS_84:NZ(HZ2) | B:SIA_1:O1B | 3.6 | 2.83 | 155.2 |
| 28 | A:LYS_84:NZ(HZ3) | B:SIA_1:O1B | 3.6 | 2.83 | 155.3 |

H-bonds were identified based on the criteria given in Extended Data Fig. 8. Distances were calculated between heavy atoms, angles were measured between donor, H, and acceptor.

**Supplementary Table 4. HKU1 N1 S1$^{A}$-ligand hydrogen bond analysis.**

| Index | Donor | Acceptor | Population | Distance | Angle |
|---|---|---|---|---|---|
| 1 | B:SIA_2:N5(HN5) | A:LYS_80:O | 95.9 | 2.93 | 155.0 |
| 2 | A:THR_82:N(H) | B:SIA_2:O8 | 83.4 | 2.92 | 158.3 |
| 3 | A:LYS_80:NZ(HZ3) | B:SIA_2:O1B | 62.8 | 2.86 | 152.6 |
| 4 | A:THR_82:OG1(HG1) | B:SIA_2:O1B | 62.0 | 2.92 | 149.5 |
| 5 | A:LYS_80:NZ(HZ3) | B:SIA_2:O1A | 54.1 | 2.98 | 140.8 |
| 6 | B:SIA_2:O8(HO8) | A:TYR_84:O | 47.5 | 2.90 | 147.2 |
| 7 | A:THR_30:OG1(HG1) | B:SIA_2:O10 | 34.3 | 2.76 | 161.4 |
| 8 | A:THR_82:OG1(HG1) | B:SIA_2:O1A | 33.8 | 2.71 | 164.1 |
| 9 | A:ASN_26:ND2(HD22) | B:9AC_3:OA9 | 20.2 | 2.92 | 157.3 |
| 10 | A:SER_246:OG(HG) | B:SIA_2:O1A | 15.7 | 2.69 | 162.7 |
| 11 | B:SIA_1:N5(HN5) | A:THR_82:OG1 | 14.9 | 3.00 | 156.3 |
| 12 | B:SIA_1:O4(HO4) | A:ASN_248:O | 14.5 | 2.81 | 156.9 |
| 13 | A:ASN_243:ND2(HD22) | B:SIA_1:O1A | 9.6 | 2.88 | 161.5 |
| 14 | A:LYS_80:NZ(HZ2) | B:SIA_2:O1B | 8.6 | 2.84 | 151.3 |
| 15 | A:ASN_243:ND2(HD22) | B:SIA_1:O1B | 5.8 | 2.90 | 159.6 |
| 16 | A:ASN_26:ND2(HD21) | B:9AC_3:OA9 | 5.1 | 2.98 | 157.1 |
| 17 | A:THR_82:N(H) | B:SIA_2:O1B | 5.0 | 2.93 | 142.9 |
| 18 | B:SIA_2:O8(HO8) | A:THR_82:O | 4.9 | 2.86 | 144.0 |
| 19 | A:THR_31:OG1(HG1) | B:SIA_2:O10 | 4.8 | 2.80 | 155.2 |
| 20 | A:LYS_80:NZ(HZ1) | B:SIA_2:O1B | 4.4 | 2.85 | 151.0 |
| 21 | A:ASN_248:ND2(HD21) | B:SIA_1:O5N | 3.4 | 2.96 | 148.6 |
| 22 | B:SIA_1:O9(HO9) | A:4YB_3:O2N | 3.3 | 2.75 | 160.0 |
| 23 | A:ASN_248:ND2(HD22) | B:SIA_1:O1B | 3.2 | 2.87 | 158.2 |
| 24 | A:ASN_248:ND2(HD22) | B:SIA_1:O1A | 3.0 | 2.87 | 158.4 |

H-bonds were identified based on the criteria given in Extended Data Fig. 8. Distances were calculated between heavy atoms, angles were measured between donor, H, and acceptor.

**Supplementary Table 5. Atom-atom contact count between HKU1 $S1^A$ and the disialoside ligand.**

| | HKU1-A Caen1 | | HKU1-A N1 | |
|---|---|---|---|---|
| **ligand-receptor contacts** | **mean** | **std** | **mean** | **std** |
| total number of contacts | 76.0 | 12.9 | 73.8 | 15.5 |
| total H-bond contacts | 6.9 | 1.7 | 8.1 | 1.9 |
| total hydrophobic contacts | 17.4 | 3.9 | 14.5 | 4.4 |
| total salt bridges | 1.4 | 0.5 | 1.0 | 0.2 |
| total favourable contacts SIA2 | 14.0 | 3.1 | 14.7 | 3.6 |
| H-bond contacts SIA2 | 5.3 | 1.3 | 6.6 | 1.4 |
| hydrophobic contacts SIA2 | 7.7 | 2.8 | 7.1 | 3.1 |
| salt bridges SIA2 | 1.0 | 0.2 | 1.0 | 0.2 |
| total 9-O-Ac favourable contacts | 7.5 | 2.4 | 5.3 | 2.2 |
| H-bond contacts 9-O-Ac | 0.6 | 0.5 | 0.3 | 0.5 |
| hydrophobic contacts 9-O-Ac | 6.9 | 2.2 | 5.0 | 2.1 |
| total SIA1 favourable contacts | 4.2 | 2.4 | 3.3 | 2.6 |
| H-bond contacts SIA1 | 1.0 | 1.0 | 1.1 | 1.1 |
| hydrophobic contacts SIA1 | 2.7 | 1.9 | 2.1 | 2.0 |
| salt bridges SIA1 | 0.5 | 0.5 | 0.0 | 0.1 |

The analysis was based on simulations starting from the *holo* state of Caen1 and N1 $S1^A$ domains in complex with the disialoside ligand with accumulated simulation times of 3 µs and 7 µs, respectively. Data were analysed in 100 ps intervals, i.e. n = 30,000 and 70,000, respectively. Potential H-bonds between matching atom pairs were identified based on a heavy atom distance threshold of 3.2 Å. For hydrophobic contacts, a distance threshold of 4 Å was used for atoms not capable of hydrogen bonding. Salt bridges refer to distances smaller than 6 Å between Lys NZ/Arg CZ and C1 atoms of the ligand. Favourable contacts (the sum of H-bond, hydrophobic and salt bridge contacts) per ligand residue are also shown in Extended Data Fig. 8.

## References


1. Li, Z. *et al.* The human coronavirus HCoV-229E S-protein structure and receptor binding. *Elife* **8**, e51230 (2019).
2. Song, X. *et al.* Cryo-EM analysis of the HCoV-229E spike glycoprotein reveals dynamic prefusion conformational changes. *Nat. Commun.* **12**, 141 (2021).
3. Walls, A. C. *et al.* Glycan shield and epitope masking of a coronavirus spike protein observed by cryo-electron microscopy. *Nat. Struct. Mol. Biol.* **23**, 899–905 (2016).
4. Yang, T. J. *et al.* Cryo-EM analysis of a feline coronavirus spike protein reveals a unique structure and camouflaging glycans. *Proc. Natl. Acad. Sci. U. S. A.* **117**, 1438–1446 (2020).
5. Tortorici, M. A. *et al.* Structure, receptor recognition, and antigenicity of the human coronavirus CCoV-HuPn-2018 spike glycoprotein. *Cell* **185**, 2279-2291.e17 (2022).
6. Yu, J., Qiao, S., Guo, R. & Wang, X. Cryo-EM structures of HKU2 and SADS-CoV spike glycoproteins provide insights into coronavirus evolution. *Nat. Commun.* **11**, 3070 (2020).
7. Guan, H. *et al.* Cryo-electron Microscopy Structure of the Swine Acute Diarrhea Syndrome Coronavirus Spike Glycoprotein Provides. *J. Virol.* **94**, e01301-20 (2020).
8. Huang, C. Y. *et al.* In situ structure and dynamics of an alphacoronavirus spike protein by cryo-ET and cryo-EM. *Nat. Commun.* **13**, 4877 (2022).
9. Kirchdoerfer, R. N. *et al.* Structure and immune recognition of the porcine epidemic diarrhea virus spike protein. *Structure* **29**, 385-392.e5 (2021).
10. Wrapp, D. & Mclellan, J. S. The 3.1-Angstrom Cryo-electron Microscopy Structure of the Porcine Epidemic Diarrhea Virus Spike Protein in the Prefusion Conformation. *J. Virol.* **93**, e00923-19 (2019).
11. Walls, A. C. *et al.* Cryo-electron microscopy structure of a coronavirus spike glycoprotein trimer. *Nature* **531**, 114–117 (2016).
12. Shang, J. *et al.* Structure of mouse coronavirus spike protein complexed with receptor reveals mechanism for viral entry. *PLoS Pathog.* **16**, e1008392 (2020).
13. Kirchdoerfer, R. N. *et al.* Pre-fusion structure of a human coronavirus spike protein. *Nature* **531**, 118–121 (2016).
14. Tortorici, M. A. *et al.* Structural basis for human coronavirus attachment to sialic acid receptors. *Nat. Struct. Mol. Biol.* **26**, 481–489 (2019).
15. Bangaru, S. *et al.* Structural mapping of antibody landscapes to human betacoronavirus spike proteins. *Sci. Adv.* **8**, eabn2911 (2022).
16. Yuan, Y. *et al.* Cryo-EM structures of MERS-CoV and SARS-CoV spike glycoproteins reveal the dynamic receptor binding domains. *Nat. Commun.* **8**, e42166 (2017).
17. Walls, A. C. *et al.* Structure, Function, and Antigenicity of the SARS-CoV-2 Spike Glycoprotein. *Cell* **181**, 281-292.e6 (2020).
18. Wrobel, A. G. *et al.* Structure and binding properties of Pangolin-CoV spike glycoprotein inform the evolution of SARS-CoV-2. *Nat. Commun.* **12**, 837 (2021).
19. Zhang, S. *et al.* Bat and pangolin coronavirus spike glycoprotein structures provide insights into SARS-CoV-2 evolution. *Nat. Commun.* **12**, 1607 (2021).
20. Wrobel, A. G. *et al.* SARS-CoV-2 and bat RaTG13 spike glycoprotein structures inform on virus evolution and furin-cleavage effects. *Nat. Struct. Mol. Biol.* **27**, 763–767 (2020).
21. Xiong Q *et al.* Close relatives of MERS-CoV in bats use ACE2 as their functional receptors. *Nature* **612**, 748–757 (2022).
22. Shang, J. *et al.* Cryo-EM structure of infectious bronchitis coronavirus spike protein reveals structural and functional evolution of coronavirus spike proteins. *PLoS Pathog.* **14**, e1007009 (2018).
23. Xiong, X. *et al.* Glycan Shield and Fusion Activation of a Deltacoronavirus Spike Glycoprotein Fine-Tuned for Enteric Infections. *J. Virol.* **92**, e01628-17 (2018).
24. Shang, J. *et al.* Cryo-Electron Microscopy Structure of Porcine Deltacoronavirus Spike Protein in the Prefusion State. *J. Virol.* **92**, e01556-17 (2018).
