# Supplementary material for: Sialoglycan binding triggers spike opening in a human coronavirus
Source: Nature. 2023 Oct 4;624(7990):201–6. doi: 10.1038/s41586-023-06599-z (PMC10700143; doi:10.1038/s41586-023-06599-z)
Supplement: Supplementary file 2 — Reporting Summary [file 41586_2023_6599_MOESM2_ESM.pdf]

nature portfolio

Corresponding author(s): Dr. Daniel L. Hurdiss
Dr. Raoul J. de Groot

Last updated by author(s): Aug 1, 2023

Reporting Summary

Nature Portfolio wishes to improve the reproducibility of the work that we publish. This form provides structure for consistency and transparency in reporting. For further information on Nature Portfolio policies, see our Editorial Policies and the Editorial Policy Checklist.

Statistics

For all statistical analyses, confirm that the following items are present in the figure legend, table legend, main text, or Methods section.

n/a	Confirmed	
☐	☒	The exact sample size (n) for each experimental group/condition, given as a discrete number and unit of measurement
☒	☐	A statement on whether measurements were taken from distinct samples or whether the same sample was measured repeatedly
☒	☐	The statistical test(s) used AND whether they are one- or two-sided<br>*Only common tests should be described solely by name; describe more complex techniques in the Methods section.*
☒	☐	A description of all covariates tested
☒	☐	A description of any assumptions or corrections, such as tests of normality and adjustment for multiple comparisons
☐	☒	A full description of the statistical parameters including central tendency (e.g. means) or other basic estimates (e.g. regression coefficient) AND variation (e.g. standard deviation) or associated estimates of uncertainty (e.g. confidence intervals)
☒	☐	For null hypothesis testing, the test statistic (e.g. F, t, r) with confidence intervals, effect sizes, degrees of freedom and P value noted<br>*Give P values as exact values whenever suitable.*
☒	☐	For Bayesian analysis, information on the choice of priors and Markov chain Monte Carlo settings
☒	☐	For hierarchical and complex designs, identification of the appropriate level for tests and full reporting of outcomes
☒	☐	Estimates of effect sizes (e.g. Cohen's d, Pearson's r), indicating how they were calculated

Our web collection on statistics for biologists contains articles on many of the points above.

Software and code

Policy information about availability of computer code

Data collection	Cryo-EM data was collected using EPU software (version 2.8.1). The MD simulations were performed using YASARA (version 22.9.24).
Data analysis	The cryo-EM maps were generated using MotionCor2, Relion (version 3.1.1), cryoSPARC live/cryoSPARC (version 4.1.0) and DeepEMhancer (version 0.13 - implemented through COSMIC2). Preliminary HKU1 model was generated using Phyre2 (version 2), and final models were built, refined and validated using UCSF Chimera (version 1.16), Coot (version 0.9.8.1), Elbow (version 1.20.1-4487), Phenix (version 1.19.2-4158) and molprobity (version 4.02-528). Analysis and visualisation was carried out with PDBePISA (version 1.52), Consurf (version 2.42), UCSF Chimera (version 1.16), UCSF ChimeraX (version 1.5), Clustal Omega (version 1.2.4) and CCP4 Superpose (version 1.0.0). Conformational Analysis Tools (CAT, www.md-simulations.de/CAT/ - version 2023) and VMD (version 1.9.2) were used for analysis and visualisation of MD trajectory data, general data processing and generation of scientific plots.

For manuscripts utilizing custom algorithms or software that are central to the research but not yet described in published literature, software must be made available to editors and reviewers. We strongly encourage code deposition in a community repository (e.g. GitHub). See the Nature Portfolio guidelines for submitting code & software for further information.

Data

Policy information about availability of data

All manuscripts must include a data availability statement. This statement should provide the following information, where applicable:

- Accession codes, unique identifiers, or web links for publicly available datasets
- A description of any restrictions on data availability
- For clinical datasets or third party data, please ensure that the statement adheres to our policy

The atomic models of the apo, holo, 1-up and 3-up HCoV-HKU1 spike have been deposited to the Protein Data Bank under the accession codes 8OHN, 8OPM, 8OPN and 8OPO. The globally and locally refined cryo-EM maps have been deposited to the Electron Microscopy Data Bank under the accession codes EMD-16882, EMD-17076, EMD-17077, EMD-17078, EMD-17079, EMD-17080, EMD-17081, EMD-17082 and EMD-17083. Data files pertaining to MD simulation results shown in Fig. 5b, and Extended Data Figs. 7-10 are made available via Zenodo under the DOI 10.5281/zenodo.7867090.

Research involving human participants, their data, or biological material

Policy information about studies with human participants or human data. See also policy information about sex, gender (identity/presentation), and sexual orientation and race, ethnicity and racism.

Reporting on sex and gender	N/A
Reporting on race, ethnicity, or other socially relevant groupings	N/A
Population characteristics	N/A
Recruitment	N/A
Ethics oversight	N/A

Note that full information on the approval of the study protocol must also be provided in the manuscript.

Field-specific reporting

Please select the one below that is the best fit for your research. If you are not sure, read the appropriate sections before making your selection.

☒ Life sciences ☐ Behavioural & social sciences ☐ Ecological, evolutionary & environmental sciences

For a reference copy of the document with all sections, see nature.com/documents/nr-reporting-summary-flat.pdf

Life sciences study design

All studies must disclose on these points even when the disclosure is negative.

Sample size

No statistical method was used to determine sample size. Sample sizes were determined by available electron microscopy time and the number of particles on electron microscopy grids. The sample size is sufficient to obtain structures at the reported resolution, as assessed by Fourier shell correlation. Three cryo-EM data sets were collected in total for this study. For the apo sample, 4207 movies were collected, 914772 particles were picked, 108396 particles went into the final global refinement and 325188 sub-particles went into the local refinement. For the holo sample, 4057 movies were collected, 956697 particles were picked. Of these, 44081 particles went into the global closed holo map, 36048 went into the 1-up map and 99174 particles went into the 3-up global structure. For the local refinements, 71458 sub-particles went into the closed holo map and 99174 sub-particles went into the local 3-up structure. For the mutant dataset, 896 movies were collected, 215843 particles were picked, 20452 went into the global refinement and 61356 sub-particles went into the local refinement.

The rationale behind the MD simulations was to simulate the conformational transitions between the two states observed by cryo-EM. In principle, it cannot be predicted beforehand how long this will take or whether it will be feasible at all with the computer resources available. The sample sizes (i.e. individual MD simulation times) for all investigated systems were chosen such that protein and ligand dynamics on the ns-µs time scale could be assessed with confidence. Accumulated simulation times are stated in the methods and range between 5 and 23 µs for the individual protein and protein-ligand systems studied. Individual simulations reached up to 1.6 µs. The total accumulated simulation time of 70 µs (for all systems) can be considered currently as very long.

Data exclusions

During cryo-EM image processing, micrographs with a CTF estimated resolution of worse than 10 Å were discarded and particles representing false picks or 'junk' particles were removed during 2D and 3D classification procedures. This is common practice for single-particle cryo-EM processing workflows.

Replication

The apo, holo and mutant datasets were collected in one session each and were not repeated. It is unattainable from time and cost to repeat cryo-EM data collection and processing on the exact same sample. For each reconstruction, two independent maps were refined in order to estimate resolution according to the recommended procedures in the field (the 'gold standard').

	Replicas of MD simulations of individual protein-ligand simulation systems are detailed in the methods (6-34 per system) and individual results can be expected in Extended Data Fig. 10. The free disialoside ligand was simulated for 10x 1µs (Extended Data Fig. 7).
Randomization	During 3D refinement of the cryo-EM structures, particles were split into two half sets. For the MD experiments, randomization is only relevant during the initialization phase of an MD simulation where initial velocities are assigned to the individual atoms according to the Maxwell–Boltzmann distribution associated with the simulation temperature.
Blinding	This study does not involve any experiments where blinding would be applicable.

Behavioural & social sciences study design

All studies must disclose on these points even when the disclosure is negative.

Study description	N/A
Research sample	N/A
Sampling strategy	N/A
Data collection	N/A
Timing	N/A
Data exclusions	N/A
Non-participation	N/A
Randomization	N/A

Ecological, evolutionary & environmental sciences study design

All studies must disclose on these points even when the disclosure is negative.

Study description	N/A
Research sample	N/A
Sampling strategy	N/A
Data collection	N/A
Timing and spatial scale	N/A
Data exclusions	N/A
Reproducibility	N/A
Randomization	N/A
Blinding	N/A

Did the study involve field work? ☐ Yes ☒ No

Reporting for specific materials, systems and methods

We require information from authors about some types of materials, experimental systems and methods used in many studies. Here, indicate whether each material, system or method listed is relevant to your study. If you are not sure if a list item applies to your research, read the appropriate section before selecting a response.

Materials & experimental systems

n/a	Involved in the study
☒	☐ Antibodies
☐	☒ Eukaryotic cell lines
☒	☐ Palaeontology and archaeology
☒	☐ Animals and other organisms
☒	☐ Clinical data
☒	☐ Dual use research of concern
☒	☐ Plants

Methods

n/a	Involved in the study
☒	☐ ChIP-seq
☒	☐ Flow cytometry
☒	☐ MRI-based neuroimaging

Eukaryotic cell lines

Policy information about cell lines and Sex and Gender in Research

Cell line source(s)	ATCC HEK-293T
Authentication	Further authentication was not performed for this study.
Mycoplasma contamination	Mycoplasma testing was not performed for this study.
Commonly misidentified lines (See ICLAC register)	No commonly misidentified cell lines were used in this study.
